# Imaging-Based Quantitative Assessment of Cage Migration After Minimally Invasive Lumbar Interbody Fusion

**DOI:** 10.3390/jcm15031069

**Published:** 2026-01-29

**Authors:** Ue-Cheung Ho, Lu-Ting Kuo

**Affiliations:** 1 Division of Neurosurgery, Department of Surgery, National Taiwan University Hospital, Taipei 100, Taiwan; coolive0510@hotmail.com; 2 Graduate Institute of Clinical Medicine, College of Medicine, National Taiwan University, Taipei 100, Taiwan

**Keywords:** cage migration, immediate cage posterior position, minimally invasive lumbar interbody fusion

## Abstract

**Background/Objectives**: Posterior cage migration is a clinically relevant complication after lumbar interbody fusion. Most reported risk factors are derived from open techniques, whereas evidence specific to minimally invasive transforaminal lumbar interbody fusion (MIS-TLIF) is limited. We evaluated factors associated with cage migration and symptomatic retropulsion in a large MIS-TLIF cohort. **Methods**: We retrospectively reviewed 650 consecutive patients undergoing MIS-TLIF, comprising 1126 fused motion segments. Cage migration was defined as posterior displacement > 3 mm compared with early postoperative radiographs. Demographic, clinical, surgical, and radiographic variables were compared between segments with and without migration. Cases with migration were further stratified by revision requirement. **Results**: Cage migration occurred in 27 of 1126 levels (2.4%). Seven cases required revision surgery for symptomatic cage retropulsion, corresponding to a level-based incidence of 0.6%. More posterior initial cage placement was significantly associated with subsequent migration. Age, body habitus, smoking, diabetes, endplate violation, and multilevel fusion were not associated with migration. Among migration cases, male sex was associated with higher odds of revision, and no radiographic or mechanical parameter predicted progression from radiographic migration to symptomatic retropulsion. In revision cases, the migrated cage was removed via the original approach, followed by contralateral placement of a new interbody cage using a minimally invasive technique. **Conclusions**: In this MIS-TLIF cohort, posterior initial cage placement was the primary factor associated with cage migration, consistent with prior open-series findings. Progression from migration to symptomatic retropulsion was not explained by mechanical parameters alone, suggesting a multifactorial process. These findings underscore the importance of meticulous cage positioning during MIS-TLIF and provide practical insights for postoperative surveillance and revision decision-making.

## 1. Introduction

Minimally invasive transforaminal lumbar interbody fusion (MIS-TLIF) has become a standard treatment for degenerative lumbar disorders, providing reduced soft-tissue trauma, decreased blood loss, and faster recovery compared with open approaches [1,2,3,4]. Despite these advantages, MIS techniques share complications seen in conventional fusion, including dural tears, neural injury, cage subsidence, cage migration, hardware failure, and pseudarthrosis [5,6,7].

Posterior cage migration (retropulsion) is an uncommon but clinically important complication after lumbar interbody fusion surgery [8,9,10,11]. When symptomatic, retropulsion may lead to recurrent radiculopathy or neurological deficit and necessitate revision surgery. Previously reported contributing factors include inadequate endplate preparation, undersized cages, posterior cage positioning, endplate injury, and insufficient fixation strength. Nevertheless, data specifically derived from MIS-TLIF cohorts remain limited, as most existing risk-factor analyses were conducted in conventional open posterior lumbar interbody fusion (PLIF) or TLIF populations [12,13,14,15]. In addition, the majority of prior studies evaluated migration at the patient level without accounting for the number of treated motion segments, and few incorporated quantitative radiographic measurements capable of characterizing early implant mechanical behavior. Revision surgery for cage migration is technically demanding due to scarring and distorted anatomy within the original surgical corridor. Re-entering the ipsilateral MIS path increases the risk of dural tears and nerve injury and may limit effective endplate re-preparation or optimal cage re-positioning [16,17,18]. Recurrent cage migration after lumbar interbody fusion has been reported in the literature [19,20]. In the present study, we describe a contralateral minimally invasive revision approach that avoids scarred ipsilateral tissue planes and recreates a clean working corridor; however, outcome data for this strategy remain limited in the existing literature. The present study analyzes a consecutive single-surgeon MIS-TLIF cohort of 650 patients (1126 fused levels), providing (1) level-based epidemiology of cage migration, (2) quantitative measurements of immediate cage posterior position (ICPP) and migration distance with reliability testing, and (3) the comparison between symptomatic migration requiring revision and conservatively managed migration in a uniform MIS cohort using a standardized cage system. Additionally, all symptomatic cases were treated using a consistent contralateral MIS revision strategy, allowing evaluation of its safety and clinical utility.

## 2. Materials and Methods

### 2.1. Participants

From our institutional database, we retrospectively identified adult patients who underwent primary minimally invasive transforaminal lumbar interbody fusion between January 2018 and December 2024 for degenerative lumbar spine disorders, including lumbar spinal stenosis, degenerative spondylolisthesis, recurrent disc herniation, and degenerative disc disease. All included patients had complete perioperative imaging data and a minimum postoperative radiographic follow-up of 12 months.

Patients were excluded if they had prior lumbar fusion at the index level, underwent surgery for trauma, infection, tumor, or deformity, or received revision fusion as the index procedure. Additional exclusion criteria included the use of mixed or non-standard implant systems, incomplete clinical or radiographic follow-up, and early reoperation within 3 months for reasons unrelated to cage migration.

After application of these criteria, 650 patients were included in the final analysis. Posterior cage migration and revision surgery were defined a priori using standardized radiographic and clinical criteria, as detailed below.

All index and revision procedures were performed by a single experienced minimally invasive spine surgeon using a standardized surgical technique. To minimize implant-related confounding, all interbody cages (straight interbody cage with a tapered tip), pedicle screws, and rods used in both index and revision surgeries were obtained from a single manufacturer, ensuring consistency in implant design, material properties, and construct stiffness across the entire cohort. This study was approved by the Institutional Review Board (IRB) of the National Taiwan University Hospital (IRB number: 202112143RINA).

### 2.2. Surgical Technique for Revision

All revision surgeries were performed using a contralateral minimally invasive approach for interbody cage reinsertion. Patients were positioned prone on a radiolucent table under general anesthesia. Target spinal levels were verified using intraoperative anteroposterior and lateral fluoroscopy.

After removal of the bilateral rods and the caudal pedicle screw on the prior operative side to facilitate tubular retractor placement, a tubular working channel was docked onto the ipsilateral facet joint. Under microscopic visualization, dense epidural scar tissue was meticulously dissected to expose the migrated cage. The implant was then securely engaged using a cage-holding device with a central locking mechanism. Once stable fixation was achieved, controlled reverse impaction was applied along the original insertion trajectory to mobilize and retrieve the cage, with continuous protection of the dural sac and traversing nerve root. In cases in which initial extraction was unsuccessful, additional mobilization was carefully performed using angled curettes and grasping instruments under direct visualization, while maintaining meticulous neural protection.

For cage reinsertion, a new tubular corridor was created through the contralateral, previously unoperated side, permitting standard MIS facetectomy and disc space preparation without scar tissue interference. Endplate preparation was performed using curettes and rasps. A Polyetheretherketone (PEEK) cage packed with local autograft mixed with synthetic bone graft substitute was inserted under fluoroscopic guidance. Supplemental bone graft material was applied anteriorly and laterally to promote circumferential fusion. Pedicle screws were reinserted percutaneously, rods were secured, and mild compression was applied to enhance cage stability. Final implant positions were confirmed via fluoroscopy.

### 2.3. Postoperative Imaging Protocol

Intraoperative fluoroscopy was routinely used to confirm the final position of both interbody cages and pedicle screw constructs. Postoperative radiographic follow-up followed a standardized imaging protocol consisting of lumbar anteroposterior (AP) and lateral radiographs obtained at postoperative day 3 and subsequently at 1, 2, 3, 6, 9, and 12 months.

### 2.4. Definition of Cage Migration

Posterior cage migration was defined as a posterior displacement exceeding 3 mm relative to the baseline position measured on the postoperative day-3 lateral radiograph, a threshold selected based on prior literature and to exceed expected radiographic measurement variability [21,22]. The distance from the posterior vertebral body margin of the caudal vertebra at the index level to the posterior cage edge was measured at each follow-up visit. Any posterior translation greater than 3 mm compared with baseline was classified as clinically significant cage migration.

### 2.5. Immediate Cage Posterior Position

Immediate Cage Posterior Position (ICPP) was defined as the linear distance (in millimeters) between the posterior vertebral body margin and the posterior edge of the cage measured on the postoperative day-3 lateral radiograph. ICPP represented the depth of initial cage placement and was evaluated as a continuous variable for association with subsequent cage migration.

### 2.6. Assessment of Endplate Injury

Initial endplate injury was defined as intraoperative or early postoperative violation of the vertebral endplate identified on fluoroscopy or immediate postoperative radiographs. Radiographic findings included visible cortical breach, focal endplate depression adjacent to the cage interface, or localized endplate irregularity suggestive of mechanical compromise. This definition was intended to capture early structural damage to the endplate distinct from delayed cage subsidence, which has been variably defined using millimeter-based height loss thresholds in prior studies. Endplate injury was recorded as a dichotomous variable.

### 2.7. Statistical Analysis

Continuous variables are expressed as mean ± standard deviation and were compared using independent-sample *t* tests. Given the markedly unequal group sizes and the potential for variance heterogeneity in subgroup analyses, Welch’s *t* test was applied when appropriate. Categorical variables are presented as frequencies and percentages and were compared using the chi-square test or Fisher’s exact test, depending on expected cell counts.

Univariate analyses were performed to compare baseline demographic, clinical, and operative parameters between patients with and without cage migration (Table 1). Patients who developed cage migration were subsequently stratified into those requiring revision surgery and those managed conservatively, and subgroup comparisons were conducted accordingly (Table 2). All statistical tests were two-tailed, and a *p* value less than 0.05 was considered statistically significant.

### 2.8. Reliability Analysis

A random sample of fused levels was selected to assess measurement reliability. Two board-certified neurosurgeons independently performed all radiographic measurements while blinded to clinical outcomes and each other’s results. Measurements were repeated after a 3-week interval in randomized order to evaluate intraobserver reproducibility. Inter- and intra-observer reliability were calculated using intraclass correlation coefficients [ICC(2,1)] derived from a two-way random-effects model with absolute agreement. ICC values were interpreted as poor (<0.50), moderate (0.50–0.75), good (0.75–0.90), and excellent (>0.90).

## 3. Results

### 3.1. Overall Cohort: Cage Migration Versus Non-Migration

Among the entire cohort, 27 patients developed posterior cage migration, whereas 623 patients showed no evidence of migration during follow-up (Table 1). Baseline demographic and clinical characteristics, including age, sex, body height, body weight, body mass index, smoking status, diabetes mellitus, and the proportion of multilevel fusion, were comparable between the two groups, with no statistically significant differences observed (all *p* > 0.05).

In patients who developed cage migration, displacement consistently involved a single operated level, irrespective of whether the index procedure consisted of single-level or multilevel fusion. No cases demonstrated simultaneous migration at multiple fused levels.

Within the non-migration group (*n* = 623), a total of 1073 motion segments were fused, comprising 36 at L2/3, 239 at L3/4, 512 at L4/5, and 286 at L5/S1. In contrast, among the 27 patients with cage migration, the affected levels included 3 at L3/4, 15 at L4/5, and 9 at L5/S1. Overall, 1126 interbody cages were implanted across the cohort of 650 patients. On a level-based analysis, the overall cage migration rate was 2.4% (27 of 1126 operated levels). Seven of these migration cases required revision surgery, corresponding to a level-based revision rate of 0.6% (7 of 1126 operated levels).

ICPP emerged as the only statistically significant mechanical determinant of cage migration. The ICPP measured on postoperative day-3 radiographs was significantly shorter in the migration group than in the non-migration group (7.2 ± 4.7 mm vs. 10.0 ± 3.4 mm, *p* = 0.004), indicating a more posterior initial cage placement in cases that subsequently developed migration. Other variables, including body height, body weight, the proportion of multilevel fusion, and the presence of endplate violation, demonstrated numerical differences between groups but did not reach statistical significance.

### 3.2. Subgroup Analysis: Revision Versus Conservative Management

**Figure 1:**
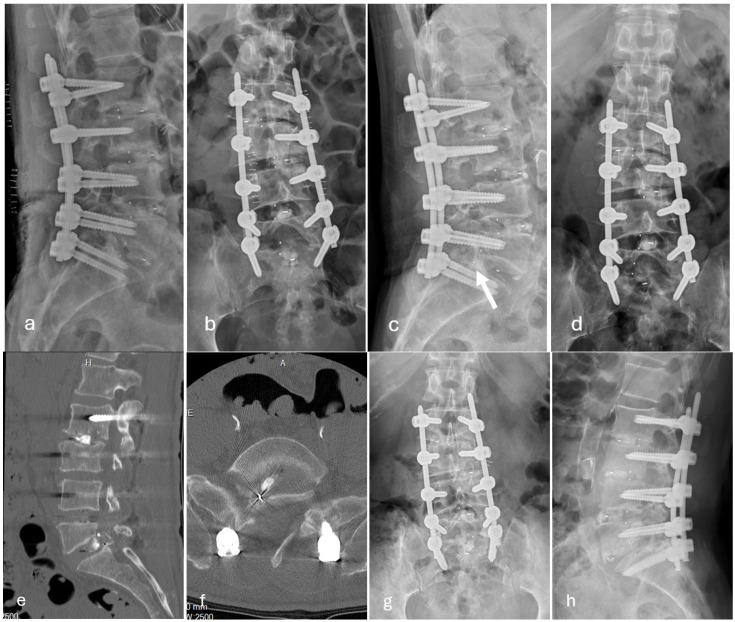
Representative imaging following minimally invasive transforaminal lumbar interbody fusion at L2/3, L3/4, L4/L5, and L5/S1. (**a**,**b**) Postoperative radiographs obtained on postoperative day 3 demonstrate appropriate interbody cage positioning at all operated levels. (**c**,**d**) Follow-up radiographs at 2 months after surgery show posterior migration of the interbody cage at the L5/S1 level (arrow). (**e**,**f**) Computed tomography images reveal posterior protrusion of the interbody cage into the spinal canal. (**g**,**h**) Post-revision radiographs demonstrate successful implantation of a new interbody cage via a contralateral left-sided approach.

**Figure 2:**
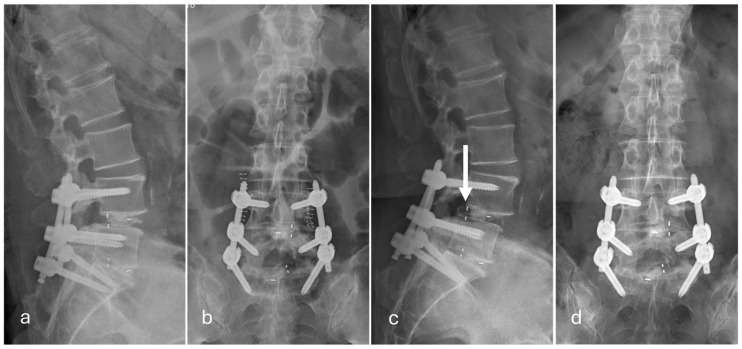
Representative radiographs following minimally invasive transforaminal lumbar interbody fusion at L4/L5 and L5/S1. (**a**,**b**) Postoperative radiographs obtained on postoperative day 3 demonstrate appropriate interbody cage positioning at both levels. (**c**,**d**) Follow-up radiographs at one month after surgery show posterior migration of the interbody cage at the L4/L5 level (arrow). No revision surgery was performed because the patient remained asymptomatic.

Among the 27 patients who developed posterior cage migration, 7 patients required revision surgery owing to symptomatic neurological deterioration (Figure 1), whereas 20 patients were managed conservatively without surgical intervention (Figure 2).

Among the seven patients who required revision surgery for symptomatic dorsal cage migration, neurological presentation was predominantly radiculopathy corresponding to the affected level. No patient presented with acute cauda equina syndrome or severe motor deficit requiring emergency intervention. In all revision cases, cage migration was progressive on interval imaging, with increasing posterior displacement observed during follow-up prior to revision surgery. Revision was performed after failure of conservative management once neurological symptoms became persistent or progressive. These findings are summarized in Table 2.

Patients in the revision group demonstrated a significantly higher proportion of males compared with those managed conservatively (85.71% vs. 45.00%, *p* = 0.049). No significant differences were observed between the two subgroups with respect to age, body height, body weight, body mass index, smoking status, diabetes mellitus, or the prevalence of multilevel fusion (all *p* > 0.05).

Cage-related and radiographic parameters, including cage height (10.9 ± 1.4 mm vs. 11.1 ± 1.3 mm, *p* = 0.76), ICPP (8.1 ± 7.4 mm vs. 7.0 ± 4.4 mm, *p* = 0.72), migration distance (12.5 ± 7.4 mm vs. 9.7 ± 4.5 mm, *p* = 0.38), and time to detection of cage migration (3.0 ± 2.2 months vs. 2.0 ± 1.9 months, *p* > 0.05) did not differ significantly between revision and non-revision patients.

Migration occurring at the lowermost instrumented vertebral level was observed more frequently in patients requiring revision surgery (100.0% vs. 61.5%), although this difference did not reach statistical significance (*p* = 0.23).

During follow-up, no patients experienced recurrent cage migration or required secondary revision surgery. Radiographic assessment confirmed solid fusion in all revision cases by one year. Clinically, all revision patients demonstrated improvement in visual analog scale and Oswestry Disability Index scores, and no major postoperative complications, including infection, dural tear, or neurological deterioration, were observed.

### 3.3. Revision Technique and Clinical Outcomes

All revision procedures were performed using a two-corridor minimally invasive strategy. The migrated cage was first removed through the original ipsilateral approach, followed by insertion of a new interbody cage through an unviolated contralateral working corridor. No case required conversion to an open procedure, and no intraoperative complications were encountered.

During postoperative follow-up, no patient experienced recurrent cage migration or required additional revision surgery. Radiographic evaluation demonstrated solid interbody fusion in all revision cases at one year. Clinically, all patients showed improvement in visual analog scale and Oswestry Disability Index scores compared with their pre-revision status. No major postoperative complications, including surgical site infection, dural tear, or neurological deterioration, were observed.

## 4. Discussion

In this series of 650 consecutive minimally invasive lumbar interbody fusion procedures involving 1126 fused motion segments, the level-based cage migration rate was 2.4%, and the revision rate for symptomatic retropulsion was 0.6%. Most migration events remained clinically silent, whereas only a minority progressed to symptomatic retropulsion requiring surgical revision. These findings emphasize that postoperative cage migration represents a spectrum of radiographic and clinical behaviors rather than a uniform failure mechanism. This reinforces the heterogeneous nature of postoperative cage migration, in which radiographic displacement alone does not necessarily indicate mechanical failure or neural compromise. To capture this variability more precisely, cage position and migration were evaluated using standardized millimeter-based measurements referenced to an early postoperative baseline, with reproducibility confirmed by inter- and intra-observer reliability testing.

A principal finding of the present study was that the ICPP was the only statistically significant factor associated with subsequent cage migration, with migrated cages demonstrating a more posterior placement at the index operation. This observation is consistent with prior large series reporting posterior cage position as one of the strongest mechanical predictors of retropulsion or migration [8,12,23,24]. However, it should be noted that the majority of these studies were based on conventional open lumbar interbody fusion techniques rather than MIS-TLIF. Differences in surgical exposure, extent of posterior element preservation, and construct stiffness between open and minimally invasive approaches may lead to distinct biomechanical environments, potentially modifying the relative contribution of cage position to migration risk. Our findings therefore refine and extend existing literature by demonstrating that posterior cage position remains a dominant risk factor specifically in MIS-TLIF. Posterior positioning reduces the anterior column load-sharing capacity and increases reliance on the weaker posterior annulus and endplate region, thereby permitting posterior displacement under axial loading. From a clinical perspective, these biomechanical considerations identify ICPP as a modifiable intraoperative parameter with direct implications for reducing migration risk.

Several factors demonstrated clinically meaningful trends despite not reaching statistical significance, warranting further discussion. Endplate violation occurred nearly twice as often in the migration group (7.7% vs. 4.1%). Although the difference did not achieve significance, some prior studies have identified endplate compromise as an important contributor to cage instability [8,23,25]. Inadequate endplate integrity reduces interface friction and subsidence resistance, predisposing the cage to posterior translation. Taken together, these findings suggest that endplate integrity remains a biomechanically relevant, albeit secondary, factor in cage stability. The relatively low prevalence of endplate violation in this cohort and the modest number of migration events likely limited statistical power for detecting an association.

Multilevel fusion also demonstrated a trend toward a higher migration rate (60.2% vs. 51.0%). Longer constructs may impose altered load-sharing mechanics and increased micromotion at individual segments, a finding supported by earlier patient-level analyses [12,14]. Although single-level and multilevel fusion were both well represented in our cohort, a nonsignificant trend toward a higher proportion of multilevel fusion was observed in patients with cage migration. This observation supports the concept that construct length may influence migration risk, particularly in the presence of other mechanical stressors. From a biomechanical perspective, this trend remains plausible, particularly when migration occurs at the lowermost instrumented level, where physiological loading and shear forces are greatest.

Body habitus has been variably implicated as a potential contributor to cage migration; however, existing evidence does not support body weight or body mass index as consistent independent predictors. In the present cohort, both parameters demonstrated numerically higher values in the migration group but failed to reach statistical significance. This finding is consistent with prior large series [8,12]. Although increased body weight may theoretically augment axial loading across the fusion construct, current evidence suggests that its influence is largely mediated through technical and biomechanical factors rather than acting as a primary driver of migration. Accordingly, body weight and BMI should be regarded as minor biomechanical modifiers rather than primary determinants of cage migration in MIS-TLIF.

Within the subgroup of patients who developed migration, the comparison between those requiring revision and those managed conservatively revealed no significant mechanical predictors of symptomatic progression. Cage height, ICPP, migration distance, endplate injury, and multilevel fusion did not differ between groups. This finding suggests that progression from radiographic migration to symptomatic retropulsion may not be determined solely by static mechanical parameters. Among revision cases involving multilevel fusion, cage migration occurred at the lowermost instrumented level in 100% of cases. Although not statistically significant, this supports the notion that the caudal level, which subjected to the highest biomechanical demand, may be more vulnerable to clinically consequential retropulsion. Male sex was the only factor significantly associated with the need for revision. Whether this reflects differences in muscle mass, physical activity, segmental loading patterns, or pain tolerance remains unclear and has not been well described in prior literature. Importantly, because earlier studies did not distinguish symptomatic from asymptomatic migration, this observation highlights a clinically relevant subgroup that warrants further investigation.

All revision surgeries in this cohort were performed using a two-corridor minimally invasive strategy. The migrated cage was removed through the original ipsilateral corridor, where neural compression had occurred, while the contralateral side was used to re-establish a clean working channel for disc preparation and insertion of the new cage. This approach minimizes the difficulties associated with scar tissue, adhesions, and distorted anatomy that commonly complicate ipsilateral re-entry after minimally invasive fusion. Prior reports describe increased risks of dural tears, nerve injury, and limited visualization when re-operating through the initial MIS corridor, underscoring these challenges [8,26].

In contrast, the contralateral working corridor provides unscarred tissue planes and more reliable anatomical landmarks, which enhance safety during decompression, endplate preparation, and cage reinsertion. In our series, this combined strategy resulted in no recurrent migration, no need for re-revision, and favorable clinical recovery in all patients. Taken together, these findings indicate that the contralateral-assisted revision technique is not only technically feasible but also clinically effective in managing symptomatic cage migration after MIS-TLIF. The technique therefore offers a practical and less morbid alternative to anterior or lateral revision procedures, which are effective but often associated with greater approach-related morbidity and longer recovery [27].

This study has several limitations. First, the number of symptomatic cage migration cases requiring revision was small, which is consistent with the low reported incidence of clinically significant retropulsion after lumbar interbody fusion. Owing to the limited number of events, multivariable regression analysis was not performed, as it could have resulted in overfitting and unreliable estimates. Accordingly, univariate analyses were used to provide transparent and interpretable assessment of potential associations. This retrospective design may also introduce selection bias and information bias related to case identification and imaging-based measurements. Second, the study was conducted at a single institution by a single surgeon using a uniform PEEK cage system, which may limit external generalizability to other surgical settings, techniques, or implant designs. Although this approach ensured procedural consistency, it may also reduce variability in surgical technique and limit extrapolation to broader clinical practice. Nevertheless, the large consecutive cohort, standardized surgical technique, and uniform postoperative surveillance protocol strengthen the internal validity of the findings, supporting their applicability to similar minimally invasive lumbar interbody fusion settings. Third, bone mineral density (BMD) was not included in the analysis because formal BMD assessment was not consistently available across the cohort. Finally, although the overall cohort was large, the number of migration events remains modest.

Overall, this study contributes several novel insights by combining level-based epidemiology, quantitative migration analysis, and the first comparison of symptomatic versus asymptomatic migration within a standardized minimally invasive fusion cohort. Larger prospective studies with standardized measurement frameworks will be needed to refine postoperative surveillance strategies, define intervention thresholds, and optimize preventive surgical techniques.

## 5. Conclusions

Our results affirm that posterior cage placement represents the dominant modifiable risk factor for cage migration, whereas progression to symptomatic retropulsion requiring revision appears multifactorial and insufficiently predicted by mechanical displacement parameters alone. The contralateral MIS revision strategy provides a technically safe and anatomically rational solution for symptomatic cases. Continued refinement of objective imaging metrics and multicenter validation studies are necessary to translate detection of migration into evidence-based clinical management pathways.

## Figures and Tables

**Table 1:** Univariate analysis comparing patients with and without cage migration.

Variable	Migration (*n* = 27)	Non-Migration (*n* = 623)	*p* Value
Age (years)	64.3 ± 12.3	64.1 ± 13.0	0.94
Sex (male)	55.5%	41.8%	0.16
Body height (cm)	163.3 ± 11.0	159.7 ± 9.4	0.11
Body weight (kg)	72.3 ± 19.2	66.2 ± 14.1	0.11
BMI (kg/m^2^)	26.8 ± 5.0	26.0 ± 4.3	0.46
Smoking (%)	18.5%	15.8%	0.73
Diabetes mellitus (%)	33.3%	31.7%	0.83
Multi-level fusion (≥2 levels)	60.3%	51.0%	0.39
Immediate cage posterior position, ICPP (mm)	7.2 ± 4.7	10.0 ± 3.4	0.004 *
Endplate violation (%)	7.7%	4.1%	0.38

* indicates statistical significance (*p* < 0.05).

**Table 2:** Comparison of migrated cages requiring revision versus conservative management.

Variable	Revision (*n* = 7)	No Revision (*n* = 20)	*p* Value
Age (years)	67.7 ± 10.8	63.1 ± 12.9	0.37
Sex (male)	85.7%	45.0%	0.049 *
Body height (cm)	169.9 ± 10.7	161.0 ± 10.4	0.085
Body weight (kg)	79.3 ± 19.2	69.9 ± 19.1	0.29
BMI (kg/m^2^)	27.3 ± 4.4	26.6 ± 5.3	0.73
Diabetes mellitus	42.9%	30.0%	0.63
Smoking	14.3%	20.0%	0.99
Multi-level fusion (≥2 levels)	42.9%	65.0%	0.36
Time to cage migration	3.0 ± 2.2	2.0 ± 1.9	0.31
Migration at lowermost instrumented level	100.0% (3/3)	61.5% (8/13)	0.23
Cage height (mm)	10.9 ± 1.4	11.1 ± 1.3	0.76
Immediate cage posterior position, ICPP (mm)	8.1 ± 7.4	7.0 ± 4.4	0.72
Migration distance (mm)	12.5 ± 7.4	9.7 ± 4.5	0.38

* indicates statistical significance (*p* < 0.05).

## Data Availability

The data presented in this study are available on request from the corresponding author. The data are not publicly available due to privacy or ethical restrictions.

## Abbreviations

The following abbreviations are used in this manuscript:

AP	Anteroposterior
BMI	Body mass index
ICC	Intraclass correlation coefficient
ICPP	Immediate cage posterior position
MIS-TLIF	Minimally invasive transforaminal lumbar interbody fusion
ODI	Oswestry Disability Index
PEEK	Polyetheretherketone
PLIF	Posterior lumbar interbody fusion
VAS	Visual analog scale
